# Emergency Use and Efficacy of an Asynchronous Teledermatology System as a Novel Tool for Early Diagnosis of Skin Cancer during the First Wave of COVID-19 Pandemic

**DOI:** 10.3390/ijerph19052699

**Published:** 2022-02-25

**Authors:** Antal Jobbágy, Norbert Kiss, Fanni Adél Meznerics, Klára Farkas, Dóra Plázár, Szabolcs Bozsányi, Luca Fésűs, Áron Bartha, Endre Szabó, Kende Lőrincz, Miklós Sárdy, Norbert Miklós Wikonkál, Péter Szoldán, András Bánvölgyi

**Affiliations:** 1 Department of Dermatology, Venereology and Dermatooncology, Semmelweis University, 1085 Budapest, Hungary; jobbagy.antal@phd.semmelweis.hu (A.J.); kiss.norbert@med.semmelweis-univ.hu (N.K.); meznerics.fanni@stud.semmelweis.hu (F.A.M.); farkas.klara@phd.semmelweis.hu (K.F.); plazar.dora@phd.semmelweis.hu (D.P.); bozsanyi.szabolcs@med.semmelweis-univ.hu (S.B.); fesus.luca@med.semmelweis-univ.hu (L.F.); lorincz.kende@med.semmelweis-univ.hu (K.L.); sardy.miklos@med.semmelweis-univ.hu (M.S.); wikonkal.norbert@med.semmelweis-univ.hu (N.M.W.); 2 Clinical Sciences Research Group, Selye János Doctoral College for Advanced Studies, 1085 Budapest, Hungary; 3 Department of Bioinformatics, Semmelweis University, 1094 Budapest, Hungary; bartha.aron@med.semmelweis-univ.hu; 4 2nd Department of Pediatrics, Semmelweis University, 1094 Budapest, Hungary; 5 Alfréd Rényi Institute of Mathematics, 1053 Budapest, Hungary; brummadza4@gmail.com; 6 MedInnoScan Research and Development Ltd., 1112 Budapest, Hungary; peter.szoldan@medinnoscan.com

**Keywords:** teledermatology, telemedicine, telehealth, COVID-19, skin cancer, melanoma, skin cancer screening, store-and-forward, diagnostic concordance, accuracy

## Abstract

Background: After the outbreak of the corona virus disease-19 (COVID-19) pandemic, teledermatology was implemented in the Hungarian public healthcare system for the first time. Our objective was to assess aggregated diagnostic agreements and to determine the effectiveness of an asynchronous teledermatology system for skin cancer screening. Methods: This retrospective single-center study included cases submitted for teledermatology consultation during the first wave of the COVID-19 pandemic. Follow-up of the patients was performed to collect the results of any subsequent personal examination. Results: 749 patients with 779 lesions were involved. 15 malignant melanomas (9.9%), 78 basal cell carcinomas (51.3%), 21 squamous cell carcinomas (13.8%), 7 other malignancies (4.6%) and 31 actinic keratoses (20.4%) were confirmed. 87 malignancies were diagnosed in the high-urgency group (42.2%), 49 malignancies in the moderate-urgency group (21.6%) and 16 malignancies in the low-urgency group (4.6%) (*p* < 0.0001). Agreement of malignancies was substantial for primary (86.3%; κ = 0.647) and aggregated diagnoses (85.3%; κ = 0.644). Agreement of total lesions was also substantial for primary (81.2%; κ = 0.769) and aggregated diagnoses (87.9%; κ = 0.754). Conclusions: Our findings showed that asynchronous teledermatology using a mobile phone application served as an accurate skin cancer screening system during the first wave of the COVID-19 pandemic.

## 1. Introduction

After the outbreak of the corona virus disease-19 (COVID-19) pandemic, restrictions were introduced all around the world in early 2020 [1,2]. Health care systems were under tremendous pressure and experienced lack of resources with the burden of the treatment of patients with COVID-19 [3]. Hospitals were also among the riskiest places where the pandemic could gain momentum [4]. For these reasons, outpatient care was restricted, most consultations were postponed for non-life-threatening conditions to prevent the spread of COVID-19 [5]. Emergency conditions were the only exceptions [6]. The use of telemedicine has come forward as an appropriate method to deal with otherwise postponed and non-urgent cases [7,8]. A patient visit where the parties only meet in an online platform, telemedicine, involves the use of various communication technologies to transmit medical data [9,10]. During the first wave of the COVID-19 pandemic, the use of telemedicine spread across different medical disciplines at an unprecedented level, including dermatology as well [11,12,13,14,15].

Different teledermatology services have been used routinely since the mid 2000’s in the public health care system of European countries, such as the United Kingdom, the Netherlands and Spain [16,17]. Despite the fact, high percentage of dermatologists used teledermatology the first time after the outbreak of the COVID-19 pandemic [18,19,20]. Teledermatology is currently used in three main ways [9]. The most widespread modality is asynchronous consultation which uses store-and-forward technologies. It relies on digital photographs of the skin lesions and history provided by the patients. Then data are transmitted to a dermatologist who performs the consultation subsequently [9,21]. The second form, synchronous consultation, which is also commonly used, allows real-time interaction between patients and dermatologists with the use of web cameras or mobile phone cameras [22,23]. The third, hybrid method, is a combination of the previous two techniques [24].

In addition to its widespread use during the pandemic, teledermatology could serve as an effective tool for the early diagnosis of skin cancer [25]. The negative impacts of COVID-19 pandemic have been already reported in the management of skin cancers [26,27]. It can only be estimated that during the first wave of the pandemic how many patients were affected by a delayed diagnosis and treatment with skin cancers [28,29,30]. In skin cancer screening, new methods of early detection has appeared during the past years due to the technical improvements of mobile phone cameras [31]. Studies were published prior to the COVID-19 pandemic that assessed the accuracy of store-and-forward type teledermatology based on photographs taken by mobile phones [32]. One additional public health aspect of store-and-forward teledermatology is that it can serve as a fast and cost-effective triage tool to reduce wait time and improve access to health care [33,34].

The aims of the present study were to evaluate the effectiveness of an asynchronous teledermatology system for skin cancer screening care and to assess aggregated diagnostic agreement.

## 2. Materials and Methods

### 2.1. Patient Data

The present study was performed at the Department of Dermatology, Venereology and Dermatooncology, Semmelweis University (Budapest, Hungary). This retrospective and single-center study included patients who submitted their cases for teledermatology consultation during the first wave of the COVID-19 pandemic, between 25 March 2020 and 13 July 2020. Asynchronous teledermatology consultations provided direct store-and-forward type visits between patients and dermatologists. To standardize the procedure, the submission of the cases was carried out by the use of a mobile phone application developed by MedInnoScan Research and Development Ltd. (Budapest, Hungary), which was made available nationwide for free of charge. The application required patients to fill in a questionnaire and send five to 10 photos of the skin lesion(s). The questionnaire included specific questions about the patients’ general health and the current complaints related to the skin lesion(s). At least one of the images had to be a photograph from a distance, to provide information about the surrounding skin adjacent to the lesion. The other images had to be close-ups, from different angles. Photos taken by patients in JPEG format could be uploaded if a resolution of 8 megapixels is achieved. The application went through continuous testing before it was released to the patients.

At the same time with the development of the application, an online interface for personal computers was created. This allowed dermatologist to view photographs and the questionnaire submitted by patients on personal computers. After uploading to the online interface, a specialist first carried out a preliminary assessment to determine if the cases required immediate teledermatology consultation. If the case was not urgent, the consultation was completed within a maximum of three business days. Then the case was treated by a specialist or a specialist registrar. Registrars were only allowed to care for patients under the supervision of a specialist. Patients were registered in the local hospital information system (HIS) (e-Medsolution, T-Systems Hungary Ltd., Budapest, Hungary) and the medical documentation was also completed there. The document included one or more possible differential diagnoses of the lesion(s) and the urgency of the case. If the dermatologists considered that the quality of the images sent by the patients were inadequate and could make it difficult to establish the correct diagnosis, it was noted as well. The medical documentation was sent to the patients by e-mail and it was also uploaded into the National eHealth Infrastructure system of Hungary (EESZT, see http://www.eeszt.gov.hu (accessed on 16 January 2022)). The EESZT provides access to data from all doctor’s health-related visits in public health institutions nationwide.

For the current study, medical records of teledermatology consultations were reviewed to obtain information on patient demographics, diagnosis, and urgency of cases who were sent for personal dermoscopy examination. Follow-up of patients was performed between 1 March and 30 April 2021, to collect the results of face-to-face (FTF) or potential histopathological examinations. Data were collected from the HIS and the EESZT. If relevant information was not found, patients were contacted by phone.

### 2.2. Diagnostic and Triage Groups

When disease categories were established, all types of malignant melanoma (MM) were placed into one diagnostic group. Similarly, all forms of basal cell carcinomas (BCC) and squamous cell carcinomas (SCC) were placed into one group, respectively. In our study, actinic keratoses (AK) were listed as a malignant lesion as well. The category of other malignancies included skin cancers that could not be accurately identified as MM, BCC, SCC, or AK. Considering non-malignant lesions, dysplastic naevi are represented as a separate group, while all other naevi were grouped together as “naevi”. Seborrheic keratoses (SK), haemangiomas and warts were defined as separate groups. All additional diagnoses with lower numbers of cases were grouped into the “other lesions” group. Table 1 summarizes the diagnostic groups involved in the study.

**Figure 1:**
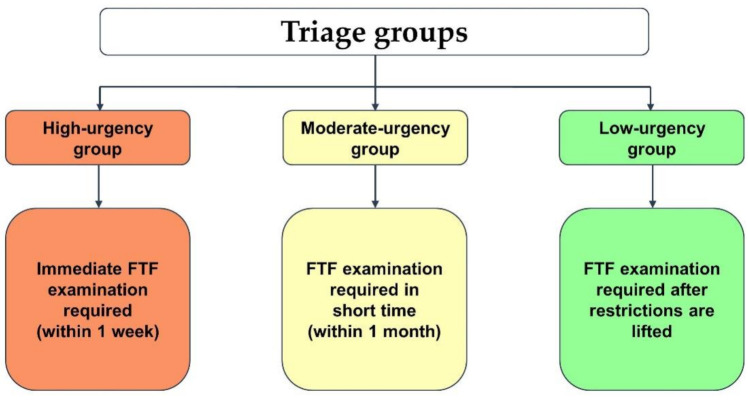
Definition of the triage groups. Abbreviations: FTF, face-to-face.

All cases were classified into three different triage groups by the immediacy of the findings during teledermatology consultations: high-urgency group (immediate FTF examination required), moderate-urgency group (FTF examination in short time) and low-urgency group (FTF examination can be postponed after pandemic) (Figure 1). If more than one lesion was referred to teledermatology consultation by the same patient, these cases received only one single triage status. In our study, this triage status was assigned to both lesions, equally.

### 2.3. Outcome Measures

The primary outcome measure was to determine the aggregated diagnostic agreement of the various diagnostic groups and the overall system, and to determine the efficacy of store-and-forward teledermatology as a skin cancer screening tool. Aggregated diagnostic agreement is defined as agreement of the primary diagnosis or any of the differential diagnoses considered during teledermatology consultation with the results of FTF dermoscopy examination or histopathology [35]. Distribution of true positive and false negative diagnoses were also evaluated among different triage groups, based on aggregated diagnosis of the lesions. Overall primary diagnostic agreement was also measured, which is the agreement between the primary diagnosis during teledermatology consultation and the results of FTF or histology examination [36].

### 2.4. Statistical Analyses

Descriptive statistics were reported for patient demographics and lesion localizations. Pearson’s chi-square test (two-tailed) was used for categorical variables. Contingency tables were used to determine diagnostic parameters, such as sensitivity, specificity, positive predictive value (PPV) and negative predictive value (NPV) of the different diagnostic groups. Cohen’s kappa coefficient (κ) was assessed for each diagnostic group separately to determine the concordance between the diagnosis established during teledermatology consultation and the reference standard. Based on the guideline, κ ≤ 0.2 indicates slight agreement, κ of 0.21–0.40 indicates fair agreement, κ of 0.41–0.60 indicates moderate agreement, κ of 0.61–0.8 indicates substantial agreement, while κ of 0.81–1.00 indicates almost perfect agreement [37]. Overall diagnostic accuracy and concordance were calculated separately for all malignant and non-malignant lesions, as well as for all lesions included in the study. Confidence intervals (95% CI) were calculated whenever appropriate. Statistical analyses were performed using Statistica v13.5.0.17 software (TIBCO Software Inc., Palo Alto, CA, USA). κ values were assessed using GraphPad QuickCalcs calculator (GraphPad Software Inc., San Diego, CA, USA).

Two reference standards were defined in this study. For lesions from which histology was performed after the FTF examination, histology was the reference standard. Where no histology was performed, the reference standard meant the result of the FTF examination.

### 2.5. Inclusion Criteria

All patients whose photographs were deemed of sufficient quality by dermatologists during teledermatology consultations and attended personal dermoscopy examination were included in this study. An additional criterion was the availability of results from the FTF examination or histology if the latter was performed.

### 2.6. Exclusion Criteria

Patients were excluded if the dermatologists could not determine the diagnosis during teledermatology consultation due to lack of proper photographs or absence of medical history. Patients were also excluded from the study if no follow-up information could be obtained.

## 3. Results

### 3.1. Patient Data

1447 patients with 1495 lesions were sent for dermoscopy examination via store-and-forward teledermatology consultation. 124 patients with 124 lesions were excluded before follow-up because of the low quality of the photographs. In 194 cases, the results of the personal examinations were found in the HIS. In 200 cases, the data were found in the EESZT. To obtain additional information, 929 patients were contacted by phone. After the follow-up, 83 patients with 83 lesions were excluded due to lack of information. 491 patients with 509 lesions did not attend FTF examination and consequently they were also excluded.

A total of 749 patients with 779 lesions were included in our study as they underwent FTF examination. Patient characteristics are shown in Table 2. The mean age of the included patients was 43.54 ± 21.03 years. The median number of lesions per patient was one (interquartile range: 1–2). 45 patients (6%) had skin cancer in the personal history, 18 patients (2.4%) had in the family history, while 2 patients (0.3%) had in both. The remaining 684 patients (91.3%) had no skin cancer in their medical history. During teledermatology consultation sessions, 639 (82%), 132 (17%), and 8 lesions (1%) received a single, two and three diagnoses, respectively. Teledermatology consultations were carried out by 29 dermatologist specialists and specialist registrars. The average number of completed cases per dermatologist was 25.8 ± 17.8. Specialist registrars, under the supervision of a specialist, cared for 565 patients with 586 lesions (75.2%). 184 patients with 193 lesions (24.8%) were managed exclusively by a specialist.

### 3.2. Triage Groups

**Figure 2:**
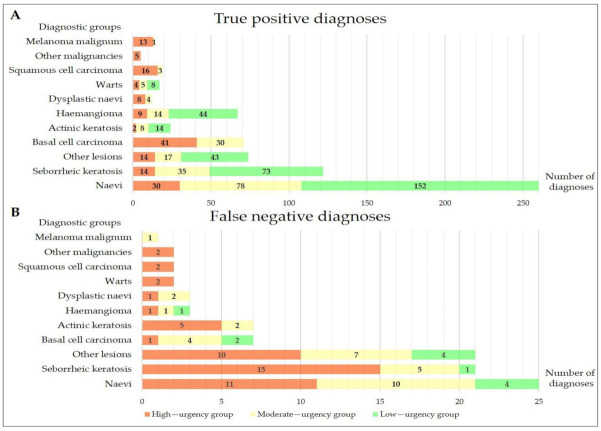
Distribution of different triage groups among true positive (**Panel A**) and false negative diagnoses (**Panel B**), considering aggregated diagnoses of the lesions during teledermatology consultations.

Figure 2 shows the distribution of triage groups among true positive and false negative diagnoses. 206 lesions (26.5%) were triaged as high-urgency, 227 lesions (29.1%) as moderate-urgency, and 346 lesions (44.4%) as low-urgency during teledermatology consultations, respectively. Two separate lesions were referred to teledermatology consultation by the same patient in 30 cases. The same triage status was assigned equally to both lesions. 

Significant differences were found between different triage groups in the number of true positive diagnoses (*p* < 0.0001). 156 lesions (75.7%) were diagnosed correctly in the high-urgency group, 195 (85.9%) lesions in the moderate-urgency group and 334 lesions (96.5%) in the low-urgency group, considering aggregated diagnoses. The reference standard was the result of the FTF examination in 509 cases and the histological examination in 270 cases. The distribution of confirmed malignancies showed also significant differences between triage groups. After the follow-up, 87 cases of malignancies were confirmed in the high-urgency group (42.2%), 49 malignancies in the moderate-urgency group (21.6%) and 16 malignancies in the low-urgency group (4.6%), respectively (*p* < 0.0001) (Figure 2).

### 3.3. Overall Primary and Aggregated Diagnostic Agreement

Table 3 reveals the overall primary and aggregated diagnostic agreement of malignant lesions, non-malignant lesions and all lesions. In terms of all malignant lesions, diagnostic concordance indicated substantial agreement. In contrast, higher concordance values were calculated for non-malignant lesions as they showed almost perfect agreement considering primary diagnosis, while aggregated diagnostic concordance indicated substantial agreement. The overall concordance between all teledermatology consultations and the reference standard showed substantial agreement. In total, significant difference was found between overall aggregated and primary diagnostic accuracy (*p* < 0.0001) (Table 3). The primary diagnosis of 633 lesions during teledermatology consultations matched with the reference standard, while 146 lesions were misdiagnosed. When all differential diagnoses were included, 685 lesions matched with the reference standard and 94 lesions did not.

### 3.4. Diagnostic Groups

The possibility of a malignancy was considered in 198 lesions based on primary diagnosis, while it increased to 228 lesions, according to aggregated diagnosis. In case of 12 lesions, the chance of two different malignancies was raised by teledermatologists. Altogether, 152 patients (male/female: 78/74; mean age: 62.26 ± 16.13 years) were diagnosed with malignancy after personal examination. Histology examination confirmed the diagnosis of MM in 15 cases, BCC in 78 cases, SCC in 21 cases, other malignancies in seven cases and AK in three patients. In case of the remaining 28 AK diagnoses, the reference standard was the result of the FTF examination. After histological examination, the following malignancies were confirmed in the other malignancies group: metastasis of Merkel cell carcinoma (1 lesion), metastasis of adenocarcinoma (1 lesion), dermatofibrosarcoma protuberans (1 lesion), invasive mammary carcinoma (1 lesion), primary cutaneous follicle center lymphoma (3 lesions). In case of malignant lesions, sensitivity values ranged from 64.5% to 89.7% (κ = 0.410–0.770) according to primary diagnoses. Sensitivity values improved to 71.4–93.3% (κ = 0.485–0.714), considering aggregated diagnoses (Table 4). Among all cases of confirmed malignancies, 24 patients (15.8%) had a history of previous skin cancer, while 6 patients (3.9%) had at least one close relative diagnosed with skin cancer. 113 patients (80.3%) did not mention any related information during teledermatology consultation.

Considering non-malignant lesions, histology examination was the gold standard in 146 cases, while FTF examination confirmed the diagnosis in 481 cases. In our study, the naevi diagnostic group included the largest number of lesions during teledermatology consultations. Concordance of naevi indicated almost perfect agreement, while the lowest agreement was assessed for dysplastic naevi, fair and moderate agreements were assessed. In case of non-malignant pigmented lesions, sensitivity values ranged from 80% to 91.2% (Table 5).

Concordance of SK (κ = 0.780) and other lesions diagnostic groups (κ = 0.676) indicated substantial agreement. Considering non-malignant diagnostic groups, other lesions had the lowest sensitivity (77.9%) as 15 out of 21 false negative cases were diagnosed as malignant lesions during teledermatology consultations. SK were misdiagnosed as malignant lesions in 14 out of 21 false negative cases. In terms of haemangiomas (κ = 0.961) and warts (κ = 0.943), almost perfect agreement was indicated (Table 6). Distribution of diagnoses of the other lesions diagnostic group is shown in the Supplementary materials (Figure S1).

## 4. Discussion

After the outbreak of the COVID-19 pandemic, there was a sudden increase in the demand for teledermatology care [38]. Temiz et al. (2020) were the first to propose that teledermatology could serve as a suitable method for the reduction of FTF consultations during the pandemic [39]. When teledermatology was used prior to the pandemic, photographs were most commonly taken by healthcare professionals using a digital camera or mobile phone [40,41,42,43]. There are just a few publications in the literature of the use of store-and-forward teledermatology consultations where photos were provided by the patients with a mobile phone’s camera and application [44,45,46].

We established a novel store-and-forward teledermatology service at our department to maintain the outpatient dermatology care during COVID-19 pandemic. Upon retrospective analysis of our data, the overall diagnostic concordance indicated substantial agreement (87.9%, κ = 0.754) between teledermatology consultations and the reference standard. In comparison, Moreno-Ramirez et al. (2007) showed an almost perfect agreement (κ = 0.81) in 890 patients [36]. Similarly, Kroemer et al. (2011) reached almost perfect agreement (90%, κ = 0.84) with the combined use of teledermoscopy and macroscopic images in 80 patients with 104 lesions [47]. Lamel et al. (2012) reported substantial agreement between FTF and mobile phone teledermatology evaluation as primary diagnostic concordance was 0.60, while the aggregated diagnostic concordance was 0.62 in 87 patients with 137 lesions [48]. Clarke et al. (2021) used macroscopic digital images taken during FTF examinations which were later sent for teledermatology assessment. They reported moderate agreement (66.6%, κ = 0.60) for primary diagnosis of 308 lesions. Based on these reports in the literature, one can conclude that store-and-forward method where the image acquisition is carried out by the patients display similar concordance to other types of teledermatology.

In previous studies, overall diagnostic accuracy for malignancies varied between 51% and 87.3%, while concordance ranged from moderate to substantial agreement (κ = 0.41–0.63) [49,50,51]. We observed a diagnostic accuracy at the higher end by reaching 85.3%, while the diagnostic concordance was 0.644. Regarding the concordance of different malignancies, we found moderate agreement for MM (κ = 0.475) and SCCs (κ = 0.560), while substantial agreement was seen for BCC (κ = 0.714) and AK (κ = 0.739). This could be related to the fact that higher number of false positive than true positive values were found in cases of MM and SCC (Table 4). Our findings showed similarities with results published by Giavina-Bianchi et al. (2020) [49]. They were using asynchronous technology to screen the population in Brazil before the outbreak of the COVID-19 pandemic and assessed consistent concordances in case of MM (κ = 0.209), SCC (κ = 0.627), BCC (κ = 0.680) and AK (κ = 724).

When all differential diagnoses were considered, high sensitivity was assessed for MM (93.3%), SCC (90.5%) and BCC (91%), similar to data in previous studies [32,47]. From a clinical perspective, false negative diagnosis of MM is a more crucial problem than it is for SCC or BCC [51]. In our set of patients, one nodular melanoma was misdiagnosed as haemangioma during teledermatology consultation. Nevertheless, triaged as a moderate-urgent case, the patient attended FTF examination within a month and the tumor was excised. This case highlights that teledermatologists should be careful with newly developed nodular vascularized or pinkish lesions. It has been shown that diagnostic of non-melanoma skin cancers (NMSC) via teledermatology is significantly hampered if the lesion is at intimate body sites [52]. In addition, in our study, one of the misdiagnosed SCC was localized in the genital region. Other studies have confirmed that the use of dermoscopic images or the combination of macroscopic and dermoscopic images for teledermatology consultations increased the diagnostic accuracy both for pigmented lesions and NMSC [53,54,55]. Contrary to our findings, malignant lesions other than MM, SCC, BCC or AK have been rarely seen in other publications [36,51]. In our study, the diagnosis of other malignancies was correctly established during teledermatology evaluation in five patients, in four of whom the availability of medical history aided the diagnosis. All these malignancies were eventually found to be recurrent tumors. Similarly to our data, Knudsen et al. have emphasized the importance of various telemedicine modalities in the follow-up of oncology patients during the pandemic [56].

Other studies have shown that teledermatology triage services for patients with suspected skin cancer can significantly reduce time to reach the diagnosis [47,57,58]. In our study, vast majority of malignancies have been categorized as to seek immediate help (Figure 2). In the low-urgency group, 14 of 16 cases of skin cancers were correctly diagnosed as AK during teledermatology consultations and the possibility of invasive skin cancer could be ruled out. Dermatologists informed these patients, taking into account the COVID-19 pandemic, that once restrictions have been lifted, they should attend FTF examination. The remaining two lesions, which were both superficial BCC, were diagnosed as SK during teledermatology consultation. Both lesions were removed with adequate safety margin after FTF, and no further treatment were needed.

The highest concordance (κ = 0.848) was calculated for naevi among pigmented lesions, while the sensitivity of this diagnostic group was 91.2%. This means that a significant proportion of non-malignant pigmented lesions have been properly recognized during teledermatology consultations. In case of dysplastic naevi, we advised immediate FTF examination to reduce the chance of misdiagnosis of MM. For this reason, concordance (κ = 0.375) for dysplastic naevi indicated only fair agreement.

In certain cases, the diagnosis of SK turned out to be challenging even during FTF examinations [59]. Furthermore, teledermatologists also consider SK as a potential false positive diagnosis for MM and NMSC [60,61]. In our study, most of the SKs were diagnosed correctly and the concordance (κ = 0.780) indicated substantial agreement that was higher compared to the results (κ = 0.513) of Giavina-Bianchi et al. (2020) [49]. Among all diagnostic groups, haemangiomas showed the highest sensitivity (95.7%) and concordance (κ = 0.961) values. In line with our findings, Betlloch-Mas et al. estimated very high concordance for haemangiomas (κ = 0.924) in the context of paediatric teledermatology care [62]. All the infants with large haemangiomas in our study were referred to FTF examination in a short term to start propranolol treatment and to rule out internal vascular malformations (Figure 2).

Our study has certain limitations. No dermoscopy images could be taken during submission of the cases. The quality of the photographs could be highly variable as they were provided by the patients. Objective inclusion criteria were not determined for the quality of the photographs and it was decided by the dermatologist if they were of sufficient quality. Further diagnostic skin imaging modalities such as high-frequency ultrasonography, optical coherence tomography and reflectance confocal microscopy were not applied for the diagnosis of skin lesions in the present study. In comparison with others, the present study was a real crisis situation setting, so it could not contain management agreement and inter-observer concordance [48,51,63]. Important outcomes such as satisfaction and costs were not addressed in this study. Some studies have excluded patients under the age of 18, while we did not. This could result in higher diagnostic parameters in the diagnosis of skin cancers and other non-malignant lesions [64].

## 5. Conclusions

To the best of our knowledge, our study was the first to investigate the efficacy of asynchronous teledermatology for the detection of skin cancer in the Central European region. Even though previous plans existed, the outbreak of COVID-19 accelerated our work and allowed real life implementation shortly after the lockdown that also restricted access to doctor’s visits in Hungary. Our findings showed that asynchronous teledermatology using mobile phone application served as a fast and accurate triage system and provided effective skin cancer care during the first wave of the COVID-19 pandemic. Teledermatology contributed to the reduction of the burden of the health system by minimizing outpatient visits and to decrease the risk of acquiring COVID-19 infection.

## Figures and Tables

**Table 1:** List of diagnostic groups.

Diagnostic Groups
Malignant melanoma
Squamous cell carcinoma
Basal cell carcinoma
Other malignancies
Actinic keratosis
Dysplastic naevi
Naevi
Seborrheic keratosis
Haemnagioma
Warts
Other lesions

**Table 2:** Characteristics of the included patients.

Variables	No.
Age composition	
0–19	87 (11.6%)
20–39	225 (30.0%)
40–59	245 (32.7%)
60–79	164 (21.9%)
80≤	28 (3.7%)
Sex	
Female	474 (63.3%)
Male	275 (36.7%)
Ethnicity	
Caucasian	744 (99.3%)
Others	5 (0.7%)
Lesion location	
Head/neck	193 (24.8%)
Hand/arm	117 (15.0%)
Trunk	350 (44.9%)
Leg/foot	103 (13.2%)
Buttock/groin	16 (2.1%)

Abbreviations: No., number.

**Table 3:** Overall diagnostic agreement.

PD/AD	Accuracy (95% CI)	Cohen’s Kappa (95% CI)
Malignant lesions		
PD	86.3% (84.1–88.7%)	0.647 (0.574–0.720)
AD	85.3% (82.9–87.9%)	0.644 (0.572–0.716)
Non-malignant lesions		
PD	81.3% (78.6–84.0%)	0.811 (0.790–0.830)
AD	86.5% (84.1–88.9%)	0.790 (0.769–0.810)
Total lesions		
PD	81.2% (78.4–83.8%)	0.769 (0.747–0.792)
AD	87.9% (85.5–90.0%)	0.754 (0.722–0.776)

Abbreviations: PD: primary diagnostic, AD: aggregated diagnostic, CI: Confidence Interval.

**Table 4:** Diagnostic parameters and concordance of malignant diagnostic groups.

PD/AD	No. of Diagnoses during TDC	TP	FN	Cohen’s Kappa (95% CI)	Sensitivity (95% CI)	Specificity (95% CI)	PPV (95% CI)	NPV (95% CI)
Malignant melanoma								
PD	32	10	5	0.410 (0.231–0.589)	66.7% (41.7–84.8%)	97.1% (95.7–98.1%)	31.3% (18.0–48.6%)	99.3% (98.44–99.7%)
AD	42	14	1	0.476 (0.317–0.636)	93.3% (70.2–99.7%)	96.3% (94.8–97.5%)	33.3% (21.0–48.5%)	99.9% (99.2–100.0%)
Squamous cell carcinoma								
PD	36	13	8	0.437 (0.273–0.600)	61.9% (40.9–79.3%)	97.0% (95.5–98.0%)	36.1% (22.5–52.4%)	98.9% (97.9–99.5%)
AD	45	19	2	0.560 (0.415–0.704)	90.5% (71.1–98.3%)	96.6% (95.0–97.7%)	42.2% (29.0–56.7%)	99.7% (99.0–100.0%)
Basal cell carcinoma								
PD	98	70	8	0.770 (0.698–0.842)	89.7% (81.1–94.7%)	96.0% (94.3–97.2%)	71.4% (61.8–79.4%)	98.8% (97.7–99.4%)
AD	112	71	7	0.714 (0.638–0.789)	91.00% (82.6–95.6%)	94.2% (92.2–95.7%)	63.4% (54.2–71.7%)	99.00% (97.9–99.5%)
Other malignancies								
PD	8	5	2	0.663 (0.386–0.941)	71.4% (35.9–94.9%)	99.6% (98.9–99.9%)	62.5% (30.6–86.3%)	99.7% (99.1–99.9%)
AD	8	5	2	0.663 (0.386–0.941)	71.4% (35.9–94.9%)	99.6% (98.9–99.9%)	62.5% (30.6–86.3%)	99.7% (99.1–100.0%)
Actinic keratosis								
PD	25	20	11	0.704 (0.566–0.842)	64.5% (47.0–78.9%)	99.3% (98.4–99.7%)	80.0% (60.9–91.1%)	98.5% (97.4–99.2%)
AD	33	24	7	0.739 (0.617–0.862)	77.4% (60.2–88.6%)	98.8% (97.7–99.4%)	72.7% (55.8–84.9%)	99.1% (98.1–99.5%)

Abbreviations: PD: primary diagnostic, AD: aggregated diagnostic, No.: number, TDC: teledermatology consultations, TP: true positive, FN: false negative, CI: confidence interval, PPV: positive predictive value, NPV: negative predictive value.

**Table 5:** Diagnostic parameters and concordance of dysplastic naevi and naevi groups.

PD/AD	No. of Diagnoses during TDC	TP	FN	Cohen’s Kappa (95% CI)	Sensitivity (95% CI)	Specificity (95% CI)	PPV (95% CI)	NPV (95% CI)
Dysplastic naevi								
PD	38	12	3	0.437 (0.270–0.605)	80.0% (54.8–93.0%)	96.6% (95.1–97.7%)	31.6% (19.1–47.5%)	99.6% (98.8–99.9%)
AD	46	12	3	0.375 (0.220–0.530)	80.0% (54.8–93.0%)	95.50% (93.9–96.8%)	26.10% (15.6–40.3%)	99.6% (98.8–99.9%)
Naevi								
PD	265	248	37	0.848 (0.809–0.887)	87.0% (82.6–90.4%)	96.6% (94.6–97.8%)	93.6% (90.0–96.0%)	92.8% (90.2–94.7%)
AD	290	260	25	0.848 (0.810–0.887)	91.2% (87.4–94.0%)	93.9% (91.5–95.7%)	89.7% (85.6–92.7%)	94.9% (92.6–96.5%)

Abbreviations: PD: primary diagnostic, AD: aggregated diagnostic, No.: number, TDC: teledermatology consultations, TP: true positive, FN: false negative, CI: confidence interval, PPV: positive predictive value, NPV: negative predictive value.

**Table 6:** Diagnostic agreement of SK, haemangiomas, warts and other lesions groups.

PD/AD	No. of Diagnoses during TDC	TP	FN	Cohen’s Kappa (95% CI)	Sensitivity (95% CI)	Specificity (95% CI)	PPV (95% CI)	NPV (95% CI)
Seborrheic keratosis								
PD	123	112	31	0.810 (0.754–0.865)	78.3% (70.9–84.3%)	98.3% (96.9–99.0%)	91.10% (84.7–94.9%)	95.30% (93.4–96.7%)
AD	154	122	21	0.780 (0.723–0.836)	85.3% (78.6–90.2%)	95.0% (93.0–96.4%)	79.2% (72.1–84.9%)	96.6% (94.9–97.8%)
					Haemangiomas			
PD	67	65	5	0.944 (0.903–0.985)	92.9% (84.3–96.9%)	99.7% (99.0–100.0%)	97.0% (89.8–99.5%)	99.3% (98.4–99.7%)
AD	69	67	3	0.961 (0.926–0.995)	95.7% (88.1–98.8%)	99.7% (99.0–100.0%)	97.1% (90.0–99.5%)	99.6% (98.8–99.9%)
Warts								
PD	15	15	4	0.880 (0.763–0.996)	78.9% (56.7–91.5%)	100% (99.5–100.0%)	100% (79.6–100.0%)	99.5% (98.7–99.8%)
AD	17	17	2	0.943 (0.865–1.000)	89.5% (68.6–98.1%)	100% (99.5–100.0%)	100% (81.6–100.0%)	99.7% (99.1–100.0%)
Other lesions								
PD	72	63	32	0.731 (0.652–0.810)	66.3% (56.3–75.0%)	98.7% (97.5–99.3%)	87.5% (77.9–93.3%)	95.5% (93.7–96.8%)
AD	111	74	21	0.676 (0.598–0.753)	77.9% (68.6–85.1%)	94.6% (92.6–96.1%)	66.7% (57.5–74.8%)	96.9% (95.2–97.9%)

Abbreviations: PD: primary diagnostic, AD: aggregated diagnostic, No.: number, TDC: teledermatology consultations, TP: true positive, FN: false negative, CI: confidence interval, PPV: positive predictive value, NPV: negative predictive value.

## Data Availability

The data that support the findings of this study are available from the corresponding author A.B., upon reasonable request.

## Supplementary Materials

The following supporting information can be downloaded at: https://www.mdpi.com/article/10.3390/ijerph19052699/s1, Figure S1: Distribution of different triage groups among true positive (Panel A) and false negative diagnoses (Panel B) of other lesions, considering aggregated diagnosis of the lesions during teledermatology consultations.
