# Supplementary material for: Emergency Use and Efficacy of an Asynchronous Teledermatology System as a Novel Tool for Early Diagnosis of Skin Cancer during the First Wave of COVID-19 Pandemic
Source: Int J Environ Res Public Health. 2022 Feb 25;19(5):2699. doi: 10.3390/ijerph19052699 (PMC8910370; doi:10.3390/ijerph19052699)
Supplement: Supplementary file 1 [file ijerph-19-02699-s001.zip › ijerph-1582126-supplementary.pdf]

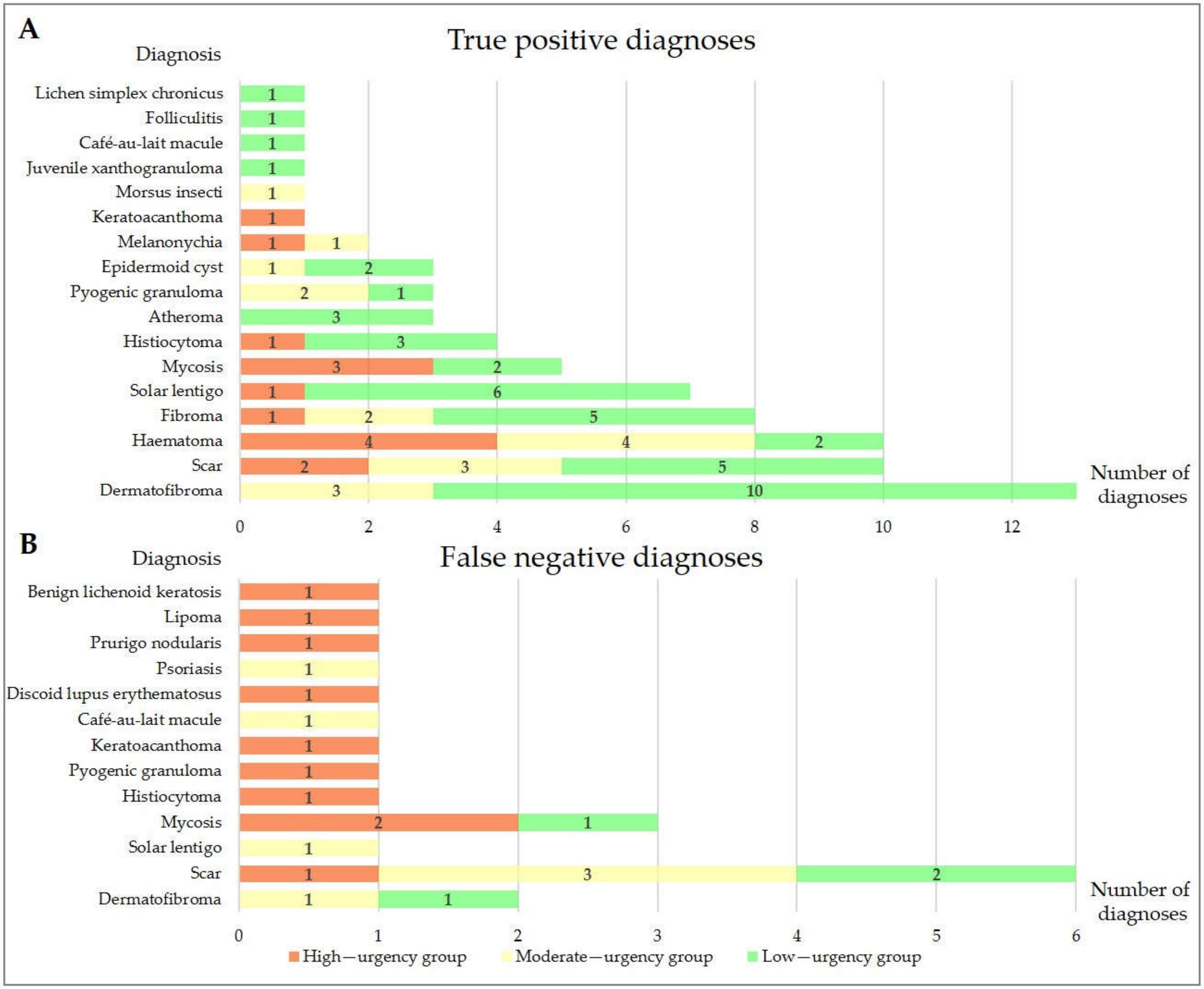

Figure S1. Distribution of different triage groups among true positive (**Panel A**) and false negative diagnoses (**Panel B**) of Other lesions, considering aggregated diagnosis of the lesions during teledermatology consultations.
